# Hanking’s Half-Yearly Abstract

**Published:** 1847-05

**Authors:** 


### RANKING'S HALF-YEARLY ABSTRACT.

We have received the second number of volume II of this valuable summary of Medical Science, and find that, so far from any falling off in its interest, the number before us is an improvement upon its predecessors. The publishers are Lindsay & Blakiston, Philadelphia, and they offer it to subscribers on terms so reasonable, ($1 50 per annum) that it is obliged to attain a most extended circulation. For this small sum, the subscriber will receive in the course of the year eight hundred closely printed pages of well digested matter, relating to all the branches of medical science.

